# Low-cost photodynamic therapy devices for global health settings: Characterization of battery-powered LED performance and smartphone imaging in 3D tumor models

**DOI:** 10.1038/srep10093

**Published:** 2015-05-12

**Authors:** Joshua Hempstead, Dustin P. Jones, Abdelali Ziouche, Gwendolyn M. Cramer, Imran Rizvi, Stephen Arnason, Tayyaba Hasan, Jonathan P. Celli

**Affiliations:** 1 Department of Physics, University of Massachusetts, Boston, MA 02125, USA; 2 Laboratoire de Physique des Lasers, Université Paris 13, 93430 Villetaneuse, France; 3 Wellman Center for Photomedicine, Massachusetts General Hospital, Boston, MA 02114, USA

## Abstract

A lack of access to effective cancer therapeutics in resource-limited settings is implicated in global cancer health disparities between developed and developing countries. Photodynamic therapy (PDT) is a light-based treatment modality that has exhibited safety and efficacy in the clinic using wavelengths and irradiances achievable with light-emitting diodes (LEDs) operated on battery power. Here we assess low-cost enabling technology to extend the clinical benefit of PDT to regions with little or no access to electricity or medical infrastructure. We demonstrate the efficacy of a device based on a 635 nm high-output LED powered by three AA disposable alkaline batteries, to achieve strong cytotoxic response in monolayer and 3D cultures of A431 squamous carcinoma cells following photosensitization by administering aminolevulinic acid (ALA) to induce the accumulation of protoporphyrin IX (PpIX). Here we characterize challenges of battery-operated device performance, including battery drain and voltage stability specifically over relevant PDT dose parameters. Further motivated by the well-established capacity of PDT photosensitizers to serve as tumour-selective fluorescence contrast agents, we demonstrate the capability of a consumer smartphone with low-cost add-ons to measure concentration-dependent PpIX fluorescence. This study lays the groundwork for the on-going development of image-guided ALA-PDT treatment technologies for global health applications.

It is well established that stark global disparities in cancer incidence and mortality exist between developed and developing countries^1^,^2^. A complex set of contributing factors are implicated including variable prevalence of risk factors such as smoking, chewing tobacco, infectious agents; limited access to sophisticated medical screening and imaging for timely cancer detection; and limited availability of cancer therapeutics in resource limited settings. In this context, we consider the potential role of photodynamic therapy (PDT), a light-based treatment modality in which wavelength-specific activation of a photosensitizing molecule that accumulates selectively in malignant tissue is used to provide site-directed tumour destruction^3^.

PDT is a non-thermal photochemistry-based therapy in which cytotoxic levels of singlet oxygen and other reactive species are generated in the target tissue following light activation. This is typically accomplished using a laser or broadband lamp to provide irradiances on the order of tens to a few hundred mW/cm^2^ in the optical window (600 to 900 nm). However, in the context of cancer treatment technologies suitable for resource limited settings, it is crucial to consider that electrical power is often unavailable or may not be sufficiently reliable and stable for clinical procedures. With this in mind it is significant to note that the development of high output light-emitting diodes (LEDs) capable of providing exactly the required illumination in a handheld format, powered by only a few volts from standard consumer batteries, suggests the potential for PDT with a low-cost, portable, battery-powered device. Although LED based sources for PDT and other phototherapy treatments have been adopted^4^,^5^,^6^,^7^,^8^, to the best of the authors knowledge, sources running entirely on battery power with the optimal combination of spectral properties, irradiance and light delivery options for ALA-PDT have not been described. For example, the commercial battery-powered phototherapy device we are aware of, the Warp 10^®^ (Quantum Warp Light Devices, Newark, OH), is approved for phototherapy treatment of muscle and joint pain and has been used in treatment of traumatic injury to the central nervous system^9^. This source emits 50 mw/cm^2^ at 670 nm from a 48 LED array. The peak emission at 670 however is not within a suitable range for PpIX excitation. Even if the device was available with 635 nm LEDs, the flat array design, which is highly conducive to its intended contact mode light delivery, would not be generally applicable for PDT cancer treatment at non-superficial sites typically requiring more customization in light delivery. Specifically, in this study the focus is on oral cancer, which ultimately requires coupling to a fiber and flexible oral insert for uniform delivery and ease of access to the oral cavity. Motivated by these factors, the present study utilizes a custom source, designed specifically for battery powered ALA-PDT, as discussed herein.

In addition to serving as therapeutic agents to enable PDT, photosensitizers can also serve in a multifunctional capacity, simultaneously generating tumour-specific fluorescence contrast for cancer imaging and treatment monitoring^10^. This, combined with the recent developments of fluorescence imaging applications utilizing smartphones^11^,^12^, which have become widely available and popular even in developing countries where other resources may be scarce^13^, suggests the capacity for PDT as a low-cost, portable, theranostic cancer technology for global health. Indeed, the simultaneous availability and built-in processing and image acquisition capabilities of smartphones, have led to increasing recognition of these ubiquitous mobile devices as a platform for cancer technologies for global health^14^,^15^.

Motivated by promising results of PDT for oral cancer treatment in traditional clinical settings^4^,^7^,^16^,^17^,^18^,^19^,^20^, this study specifically seeks to evaluate a technology that would extend the benefit of PDT for this form of cancer to the most resource-limited settings, with little or no access to electricity, refrigeration, or medical infrastructure. This motivation is well-founded as in addition to being conducive to PDT treatment; oral cancer is indeed a global health priority in many developing countries. In India for example, oral cancer accounts for over 30% of cancers reported due to the widespread abuse of carcinogenic chewing tobacco mixtures containing crushed betel nut and acacia extract^21^. The current treatment options, primarily surgery and/or radiation, can be curative if cancer or dysplasia is caught at a sufficiently early stage. Unfortunately, many patients do not seek medical attention until the disease has progressed to a point where radical operation is required (Stage III/IV), entailing block dissection and removal of the entire lymphatic drainage of the neck. Even after this procedure, disease often still recurs, leading to an overall survival rate of less than 70% in these cases^22^. This is in part because of lack of medical services in the rural areas where patients need them most. Even in cases where timely treatment with surgery and/or radiation is administered, it may be disfiguring, with significant impact upon quality of life. In contrast, clinical studies of PDT for oral cancer treatment collectively indicate that PDT is a safe and effective approach, with remarkable healing of the mucosa, and it is particularly effective for early stage cancerous and precancerous lesions of the oral cavity^4^,^7^,^16^,^17^,^18^,^19^,^20^.

This study focuses specifically on the challenges and trade-offs that are required for achieving robust light delivery in the wavelength, irradiance and fluence windows relevant for clinical ALA-PDT implementation, while considering component cost, weight/portability, and widespread availability of consumable components for resource limited settings. LED-based light-sources for PDT have been explored previously and shown to have no inherent disadvantage relative to a broadband clinical lamp or a laser source^4^,^7^. In fact, given the photochemistry requirements of PDT, there is no reason that a coherent light source (laser) is needed. This study, however, addresses practical challenges in achieving a consistent irradiance over the needed field of irradiation and treatment duration (total fluence delivered) that arise when relying entirely on battery power. In this study, we adopt a 3D cell culture approach as a useful and biologically relevant platform with established utility in evaluation of PDT efficacy^23^,^24^,^25^,^26^,^27^,^28^.

As with previous studies evaluating PDT treatment of oral cancer, the present study is pursued in the context of the established photosensitization strategy of aminolevulinic acid (ALA) induced protoporphyrin IX (PpIX)^29^,^30^. The favoured excitation maximum of PpIX at 635 nm for PDT corresponds to a 1.95 eV bandgap that falls in a window that can be readily achieved at low cost with aluminium gallium arsenide, gallium arsenide phosphide or other standard semiconductor materials for LED fabrication. For therapeutic activation of PpIX we evaluated a battery-powered source built on a commercially available high-output 635 nm LED, while for fluorescence imaging we implement a smartphone mounted array of 405 nm LEDs. We characterize the performance of these devices relevant to the requirements of PpIX mediated photo-destruction and imaging respectively in squamous carcinoma cells, a common cancer of the oral cavity. Using monolayer and 3D cell culture models we show strong cytotoxic response and quantitative PpIX imaging as part of an envisioned implementation of an LED-based, battery powered and fully portable image-guided PDT platform for global health applications.

## Materials and Methods

### Cell culture and reagents

A431 squamous carcinoma cells were obtained from American Type Culture Collection (ATCC, Rockville, MD) and grown according to ATCC recommendations in DMEM (HyClone, Logan, Utah) supplemented with 10% FBS (HyClone), 100 IU/mL penicillin (Life Technologies, Carlsbad, CA), and 100 μg/mL streptomycin (Life Technologies). Cell cultures were maintained at 37 ^o^C in a humidified atmosphere of 95% air and 5% CO_2_.

### 3D cell culture

A431 cells were overlaid on beds of Growth Factor Reduced (GFR) Matrigel (Corning, Tewksbury, MA) and grown for 7 days in media supplemented with 2% GFR Matrigel to form multicellular in vitro 3D nodules, as characterized extensively by ourselves and others for a variety of cancer cell lines^31^,^32^,^33^,^34^. Cultures were plated in this manner into glass bottom, black-walled 24 well plates (ibidi GmbH, Germany) for assignment into PDT treatment groups and parallel imaging based treatment assessment as described below.

### Battery-powered PDT irradiation device

The prototype battery-powered 635 nm irradiation device used in this study is built in an aluminium box with breadboard slot for electronics and a battery pack for 3 AA batteries as shown in Supplemental Figure 1. The light source used was a single high-output light emitting diode with peak emission at 635 nm and FWHM of 40 nm, built in a TO-39 housing (OptoDiode OD-624L-ND). To manage thermal energy dissipated in the diode a TO-39 star heat sink was coupled to the LED housing with thermal compound (Wakefield Thermal Solutions, Pelham, NH). Battery power supply and internal electronics were evaluated as reported in the Results. All treatment response data was obtained using 3 AA alkaline batteries connected in series to power the LED, though some validation tests were run using either a 9 V alkaline battery or an external power supply (Keithley, 2401 Source Meter). Internal electronics included a 5 Ω power resistor and voltage regulator (LM317, Fairchild semiconductor).

### LED beam profile characterization

Measurement of the LED beam profile was performed using a Keithley 2401 voltage (1 μV - 20 V and 10 pA - 1 A) source meter to regulate LED current. Output power was measured by a Newport Power Meter Model 2935-C power/ energy meter connected to an 818 Photo-detector, Silicon, 400-1100 nm, DB15 Calibration Module mounted horizontal to the light source at a fixed position on an optical table, using a pinhole aperture fixed at 1 mm for beam discretization. Distance from detector to LED is measured and is in the Z direction. The light source was mounted to an NRT100 - 100 mm Motorized Linear Translation Stage (Stepper Motor 1/4” - 20 Taps) directly in front of the power meter. Measurements were obtained by centrally positioning the light source to the photo-detector and horizontally scanning the full width of the beam using a BSC203 - Three-Channel apt TM Benchtop Stepper Motor Controller. Due to random noise, data points were collected in 5 - 10 second intervals by averaging the stabilized min and max.

### Light source and electronics performance and power measurements

LED characterization was performed using a Keithley 2401 voltage (1 μV – 20 V and 10 pA – 1 A) source meter to ramp current at controlled voltage. Output power was measured by a Melles-Griot 13PEM001 broadband (400 nm to 2 um) power/ energy meter (10 μW to 2 W) mounted vertically above the light source using a lab stand while LED temperature was monitored using a thermocouple in contact with the LED throughout irradiation. Measurements were obtained by vertically mounting the meter above the heat-sinked LED. Battery drainage with respect to LED output measurements were performed using the same setup with a multimeter (Fisher Scientific) connected across the power supply. The battery-powered device included a 3 terminal positive adjustable regulator with output voltage adjustable over a 1.2 V to 37 V range (LM-317, Fairchild Semiconductor).

### Battery powered photodynamic therapy (PDT) treatment

All PDT treatments were performed using Protoporphyrin-IX (PpIX) induced by administration of exogenous 5-Aminolevulinic-Acid (ALA) (Acros Organics, New Jersey, US). Cultures in either 24 or 96 optically clear black-walled multiwell plates (Nunc, Thermo Fisher Scientific, Verona, WI) were incubated in media containing 1 mM ALA for 4 hours. Immediately prior to 635 nm irradiation, media was replaced with complete growth media and irradiated with the specified fluence. The battery-powered device was positioned vertically under a rigid, transparent plexiglass sheet supporting the multiwell plates. The LED light source was positioned at 1 cm from the underside of the cell culture surface, measured to provide a roughly uniform irradiance of 68 mW/cm^2^ over a circular region of approximately 5.33 mm diameter at an LED power output of 15 mW. As reported here, output power does fluctuate over the life of the battery so power was checked and recalibrated (if needed) after each treatment.

### Image-based treatment assessment

In both monolayer and 3D cell culture studies, outcomes were evaluated 24 hours after treatment by an adaptation of an imaging-based methodology previously described^25^,^32^. Briefly, multiwell cultures were labelled with fluorescent vital dyes Calcein AM and Ethidium Bromide (Life Technologies, Carlsbad, CA). 5X mosaic images of 36 individual microscope fields were obtained from the central region of each well and stitched using a Zeiss AxioObserver Z.1 microscope with automated motorized stage and standard fluorescence filter cubes (Carl Zeiss) and equipped with a cooled, high-resolution monochromatic 14-bit digital camera (AxioCam, Carl Zeiss). Cultures incubated with ALA but not exposed to light were used as controls. Each individual dose group was repeated three to six times. Image data was batch processed using custom Matlab software routines adapted from a protocol previously reported to quantify viability based on ratiometric analysis of cleaved calcein and intercalated ethidium bromide fluorescence signals^32^. For the 3D cultures, total volume of residual disease was based on segmentation of fluorescence signal from cleaved Calcein AM. For each experiment the total residual volume was reported as the average over three replicates for each dose and normalized against untreated samples to evaluate PDT dose response. Additional confocal images to verify 3D morphology and depth-dependent cytotoxicity were obtained using a Zeiss LSM 510 laser scanning confocal microscopy. Confocal optical sections with 5um spacing were obtained using an Argon 488 nm laser and a 594 nm diode laser to excite cleaved calcein and ethidium bromide respectively of identically prepared and treated 3D cultures.

### Smartphone-based PpIX imaging

Titanium dioxide (TiO_2_) tissue phantom solution was created by adding 36.5 mg TiO_2_ (Titanium(IV) oxide, Acros Organics) to 50 mL PBS resulting in a 0.73 g/L concentration informed by the literature^35^. Free acid PpIX (Sigma-Aldrich, St Louis MO) was dissolved in a mixture of PBS, 1 M KOH, and 1 M HCL and then diluted to a concentration of 50 μg/mL in PBS. Varying amounts of this this stock solution were then added to 1600 μL of TiO_2_ solution and PBS was added to each sample to bring them to 2 mL. We created 3 replicates of each of 13 identical tissue phantoms (0.584 g/L TiO_2_) spanning physiologically-relevant PpIX tissue concentrations (1- 10 μg/mL)^36^. Cuvettes containing tissue phantoms with varying concentrations of PpIX were individually remixed and then imaged with the built in camera on a smartphone iPhone 5S, Apple Inc., Cupertino, CA equipped with a 405 nm LED array (Eigen Imaging Inc., San Diego, CA) and a 0.5 inch diameter 660 nm spectral filter (100 nm bandwidth, Chroma Technology) placed directly over the image sensor. A431 cultures were prepared for smartphone imaging by incubating an entire T75 culture flask in 1mM ALA for four hours prior to harvesting and centrifugation to form a pellet approximately 1 cm in diameter. ALA-treated pellets and identically prepared and harvested controls with no ALA were then imaged in the same manner as the tissue phantoms. The cell phone was secured in place so as to maintain the same object distance for each measurement. Images were analysed using custom routines developed in the MATLAB image processing toolbox.

## Results and Discussion

### LED performance and thermal management

**Figure 1:**
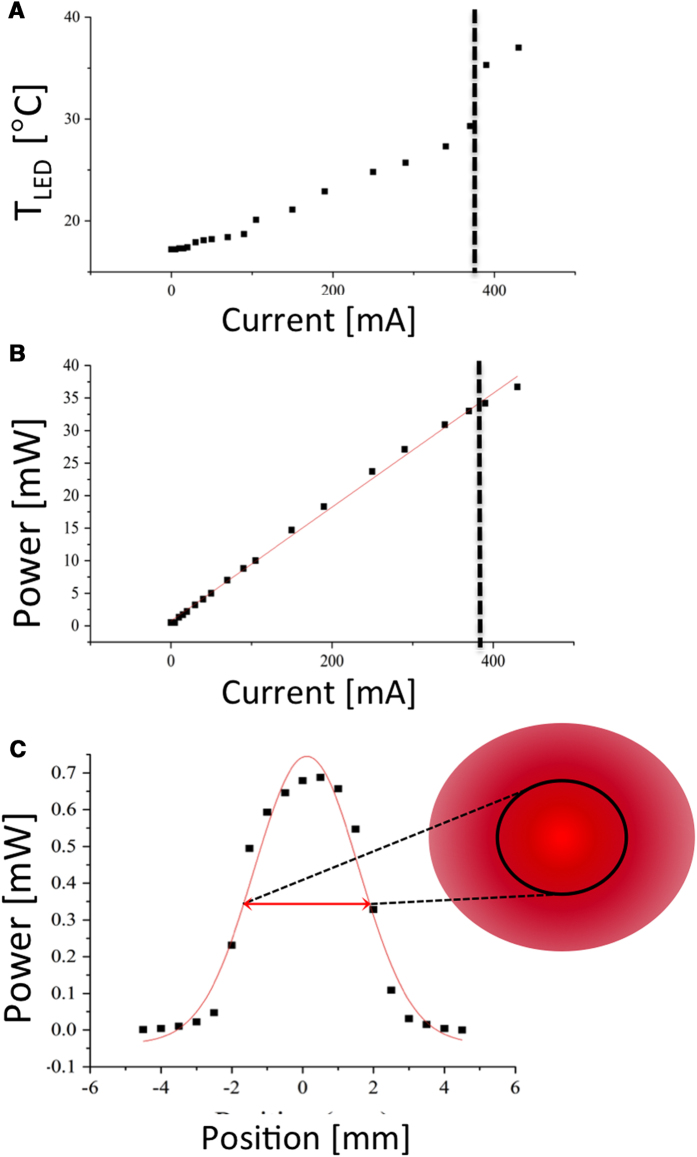
LED performance and thermal characterization. OptoDiode LED temperature (**A**) and power output (**B**) versus operating current, showing linear dependence up to the onset of thermal performance loss near 400 mA. Both measurements were performed with heat sink in an unenclosed geometry and ambient laboratory temperature. LED beam profile (**C**) with Gaussian fit and its FWHM. To the right of the plot is a projected 2D representation of the LED beam and its associated FWHM.

Before operating under battery powered we first characterized the performance of the high-output LED (OptoDiode OD-624 L) using a robust external power supply (Keithley 2401 source meter) to establish a realistic target current for our battery-powered device. As shown in Fig. 1, the irradiated power output of the LED coupled to a heat sink in ambient lab atmosphere exhibits linear dependence on current up to approximately 375 mA, which coincides with a sharp increase in LED temperature, measured simultaneously. The onset of suboptimal performance with elevated temperature is consistent with the manufacturer’s documentation on thermal derating of this LED. In the linear regime, output power, P, scales with current, I, as P = (0.09 mW/mA)×I. Further testing of LED performance housed inside an enclosure showed no significant difference. This relationship is important in defining trade-offs in irradiance (and hence treatment duration) and was subsequently used in setting the device’s operating current. We also established the beam profile (Fig. 1C) with no additional collimating optics to be approximately consistent with the manufacturer’s documentation of the angular distribution of radiation with a half angle of about 5 degrees and appropriate for treating small lesions of ~2 cm^2^ without additional optics.

### LED battery drain and power output stability under PDT-relevant operating demands

**Figure 2:**
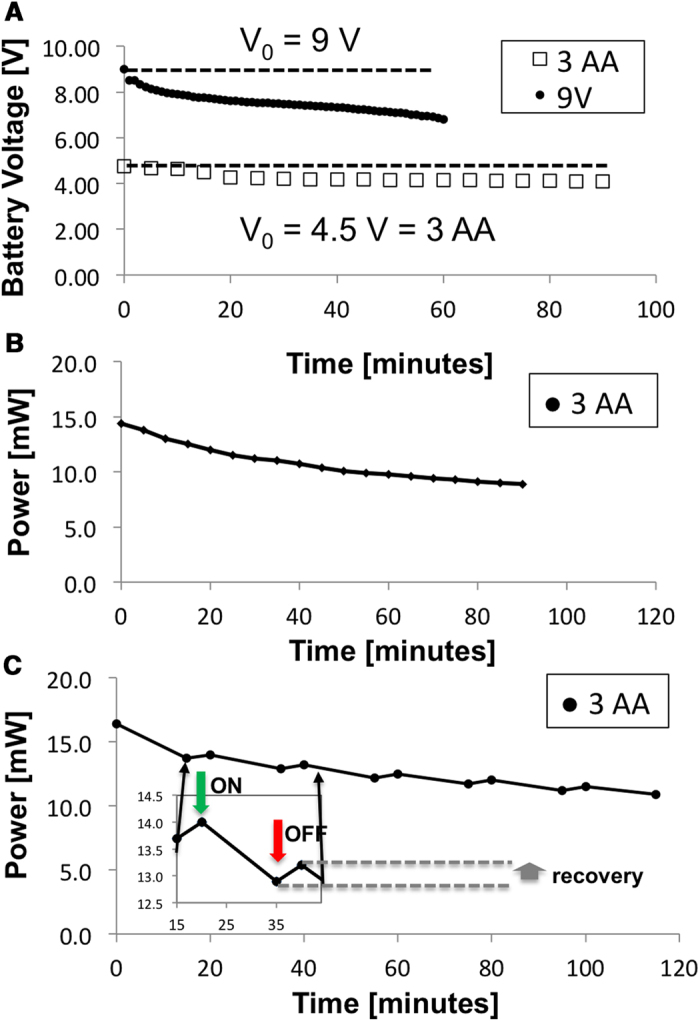
Characterization of battery-powered LED device performance. In (**A**) battery voltage over time under continuous drain with a 9 V battery source is compared to 3 AA (1.5 V) batteries in series showing a brief period of high voltage operation but rapid drain in the former, versus relatively stable output in the latter. Further characterization of LED power output over extended period of continuous drain using 3 AA batteries is shown in (**B**) In (**C**) LED power output over time for 15 minute bursts followed by 5 minute rest periods (again using the more stable 3 AA battery pack) is shown to extend battery life due to partial recovery after each burst.

Informed by LED calibration above we sought to measure performance operating under battery power. We compared voltage stability when powered by a single 9 V alkaline battery, versus a battery pack containing 3 AA alkaline batteries (Fig. 2A). Battery voltage measurements were obtained with a multimeter connected across the terminals during constant continuous voltage powering only the LED itself in series with a protective resistor providing an initial power output of 32.5 mW (9 V) or 16.4 mW (3 AA). (For the spot size used in subsequent treatment, these powers correspond to irradiances of 146 mW/cm^2^ and 74 mW/cm^2^ respectively). As shown in Fig. 2, battery drain is dramatic in the 9 V case, with a voltage decrease of 25% in 60 minutes (at which time the test was terminated) compared to a drop of only 13% over a 90 minute interval for 3 AA batteries. It is also noteworthy that battery voltage is nearly constant in the latter case after an initial drop in the first 15 minutes of continuous operation.

If the LED is operated continuously, power output also drops (Fig. 2B) though continuous operation at these power densities for 90 minutes is highly unlikely. Given a target irradiance on the order of 50 - 100 mW/cm^2^ to provide a total fluence of 20 - 100 J/cm^2^ likely to be useful for clinical PDT, we evaluated battery drainage in 15 minute bursts of continuous operation, with 5 minute rest periods shown in Fig. 2C. As expected, the battery voltage (and power output) partially recovers after each 15 minute burst, and in this case there is only a 7.5% total decrease in battery pack voltage over 6 periods of operation. If bulk and weight were not considerations, then voltage stability would presumably be even greater with size C or D batteries though the trade-off is likely not worthwhile given the performance of 3 AA batteries and the large increase in bulk going to the next incremental consumer battery size.

To determine if the error introduced into dosimetry due to this power drop over a 15 minute treatment is significant, we compare the total energy delivered over each interval assuming (the standard scenario) constant output during operation *P*_0_Δ*t*, versus an approximation of linear decrease knowing the final power of each operation interval, 
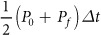
. (We note here that a more precise estimate of the energy delivery is to model *P(t)* as exponential decay, and integrate,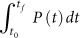, however the difference is negligible over these short bursts). In this manner we calculate the percentile error in dosimetry shown in Table 1. With an average value of 4.4%, including the largest (initial) drop, this is a source of error which is dwarfed by uncertainty in myriad other PDT dosimetry parameters including irradiation duration, patient position, background light, and photosensitizer incubation time and uptake variation. The battery voltage recovery when operating in bursts further suggests a potential practical benefit to fractionated light delivery under these circumstances, beyond the documented photophysical/photochemical enhancement of fractionated PDT^37^,^38^. This is discussed further below.

### Evaluation of device performance and effective irradiation field in monolayer cultures

**Figure 3:**
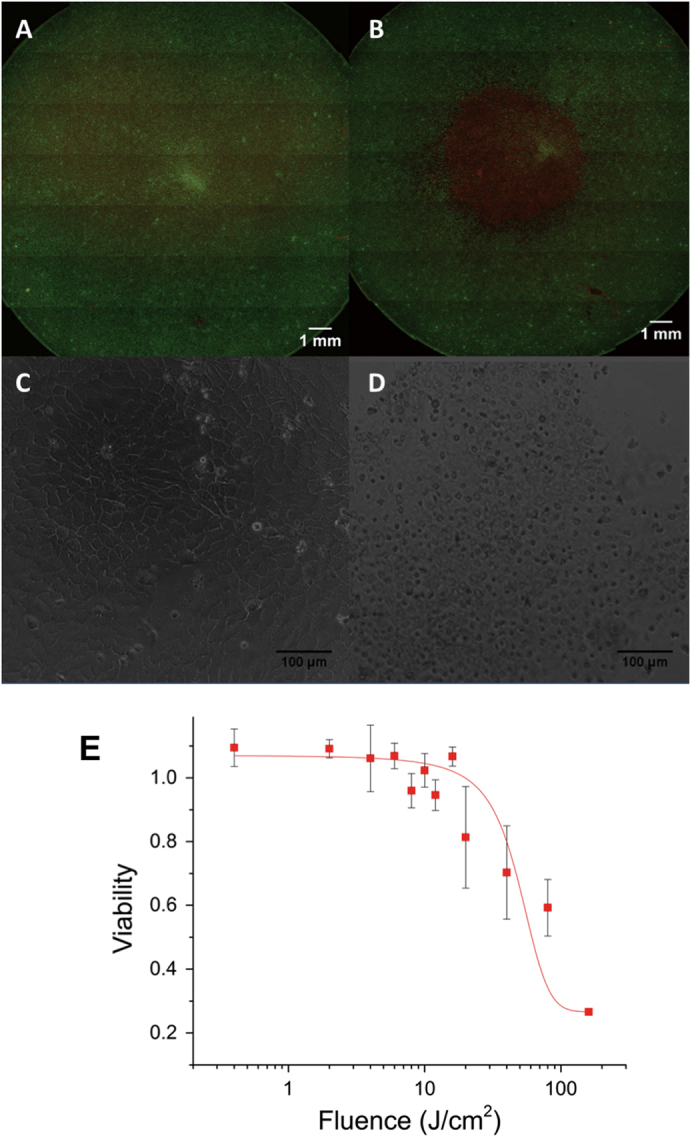
Evaluation of effective treatment area and cytotoxicity in squamous carcinoma cells. A431 squamous carcinoma cells were cultured and then treated with the battery powered LED light source. (**A**) Untreated A431 cells cultured in monolayer and stained with calcein AM as a fluorescent reporter of cell viability (green) and ethidium bromide for labelling of dead cells (red). (**B**) A treated culture, showing the pronounced zone of cell death (red) approximately matching the irradiation spot size. Phase contrast images of untreated (**C**) and treated (**D**) cultures showing obvious visual indication of cell death in the latter. A representative dose-response curve in (**E**) exhibits sigmoidal form and approaches complete killing within the sensitivity of the fluorescence measurements at doses above 10 J/cm^2^.

Using the battery-powered device operating parameters established above, we evaluated device performance for ALA-PDT in A431 human squamous carcinoma cells (Fig. 3). Cells were incubated with ALA to accumulate PpIX, irradiated with the battery-powered device, and evaluated. In oversize monolayers (relative to the spot size) we observed a consistent zone of nearly total cell death consistent with the spot diameter of approximately 5 mm (Fig. 3B) which is not present in untreated controls (Fig. 3A). Since the full culture vessel received ALA and converts PpIX, these results confirm that there is negligible toxicity due to photosensitization alone. Phase contrast images of viable cells in untreated controls compared with battery-powered PDT treated cells are shown in Fig. 3C,D respectively. Evaluation of fractional viability was performed by image-based quantification of large multi-tile fields within the irradiated spot as shown in Fig. 3E. Dose response is sigmoidal as expected, with nearly complete cytotoxic response at the highest doses assessed.

### Battery-powered PDT killing in 3D cell cultures

**Figure 4:**
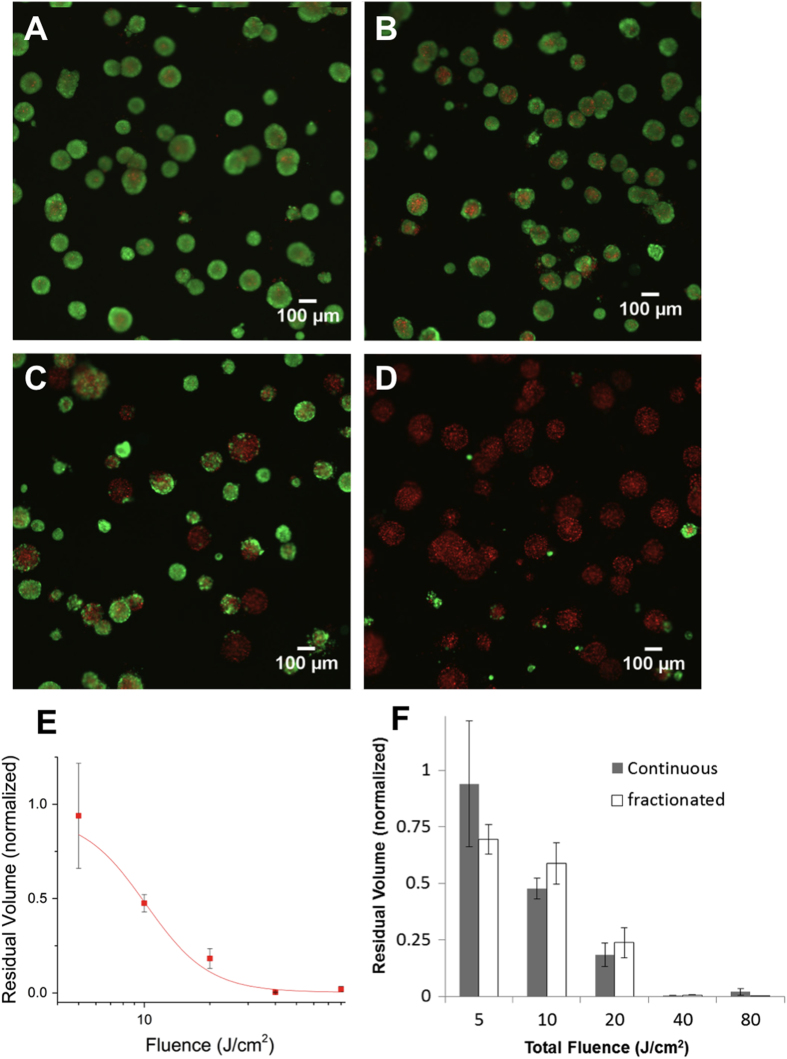
Evaluation of battery powered ALA-PDT response in 3D cell cultures. (**A**-**D**) Representative images of A431 3D cultures treated with ALA and irradiated with battery powered 635 nm source stained with vital dyes, calcein AM (green) and ethidium bromide (red) for the specified doses (A is ALA only with no light activation, **B C** and **D** are 10, 20, and 80 J/cm^2^ respectively). Increasing cytotoxicity is observed with increasing dose, with nearly complete cell death at 80 J/cm^2^. (**E**) Quantitative dose response obtained from full image datasets, in terms of total residual viable disease volume, averaged over a minimum of 3 replicates and normalized to the ALA treated control average. (**F**) Comparison of fractionated and continuous light delivery. In the former case each total fluence achieved is broken into fractions of one third total dose with 1 minute rest periods in between. Although dosimetry error is smaller, for this fractionation regimen in this cell culture model the response at each dose is not significantly different from the same dose achieved with continuous delivery (p > 0.05).

Having established the effective treatment area of the prototype device in monolayer cell culture we sought to further validate the efficacy of treatment in a more biologically relevant disease model. A431 squamous carcinoma cells overlaid on basement membrane were observed to spontaneously form multicellular 3D micronodules over the course of one week in these culture conditions. The approximately spherical nodule morphology is consistent with previous reports on 3D growth of several epithelial cancer cells^31^,^32^,^33^,^34^, is also shown in confocal 3D images in Supplemental Figure 2, in which mean nodule diameter (and approximately equivalent thickness) is measured to be 101 + /- 27 μm. 3D cultures were incubated with ALA for 4 hours to accumulate PpIX and treated with the battery powered device to achieve the total fluence indicated. As shown in Fig. 4 administration of increasing light doses (Fig. 4B-D) results in increasing cytotoxicity relative to untreated controls (Fig. 4A). Quantification of cytotoxic response (Fig. 4E) shows nearly total cytotoxic destruction of 3D tumour nodules with sufficiently high light dose. The nearly complete cytotoxic destruction throughout the full depth of 3D multicellular nodules is further characterized in Supplemental Figure 2, showing depth-resolved confocal sections of nodules greater than 100 μm in thickness. Penetration of light through this thickness is consistent for expectations of PpIX excitation using a 635 nm light which is within the optical window.

Further motivated by the improved battery performance when light delivery is fractionated into brief bursts, we evaluated administration of the same total light doses delivered in two minute fractions in Fig. 4F. Under these operating conditions there was no significant difference in therapeutic efficacy (p > 0.05 for all doses) aside from the small improvement in dosimetry accuracy due to a decreased total drain over the course of any single dose administration. However, it is likely that the therapeutic benefit of fractionated delivery with this system would be realized for larger, vascularized tumours and possibly to an extent that would outweigh the clinical drawback of increased total treatment time.

### PpIX fluorescence detection via smartphone camera

In developing a platform for PDT treatment conducive to global health implementation, it makes sense to also consider similarly suitable approaches to integrate PpIX fluorescence imaging for tumour detection and PDT treatment planning and monitoring. The availability in the developing world of smartphones with integrated cameras, recent studies that develop sophisticated smart phone imaging techniques^11^, and the inherent capability for telemedicine integration motivated us to investigate the capability of a consumer smartphone as part of this low-cost ALA-PDT platform. In PpIX tumour imaging it is well established that the broader blue-violet absorption maximum (peak at approximately 409 nm) is preferred^10^. Although attenuation by scattering an absorption in tissue is much higher, the strong absorption and well separated Stokes shift to red fluorescence emission is conducive to imaging.

**Figure 5:**
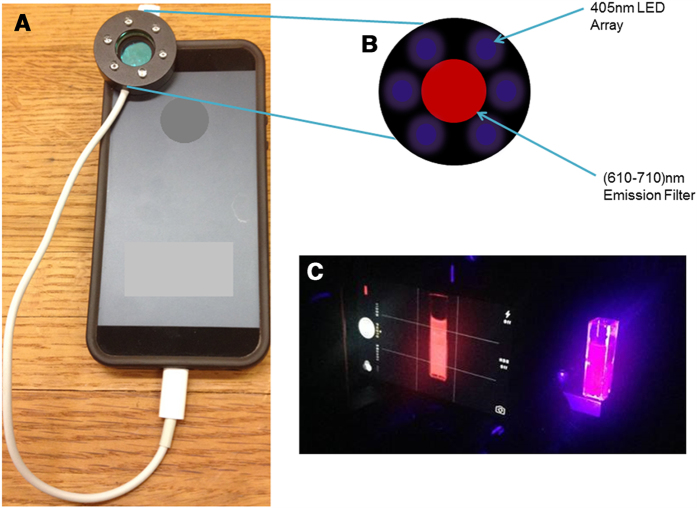
Adaptation of consumer smartphone for PpIX fluorescence imaging. (**A**) A smartphone equipped with a 405 nm LED array (modified FluoroVu device, by Eigen Imaging) fitted with a 610-710 nm emission filter, as shown schematically in (**B**) (**C**) Photograph of representative view on smartphone display, imaging a PpIX phantom excited with LED array and imaged through an emission filter over phone camera.

**Figure 6:**
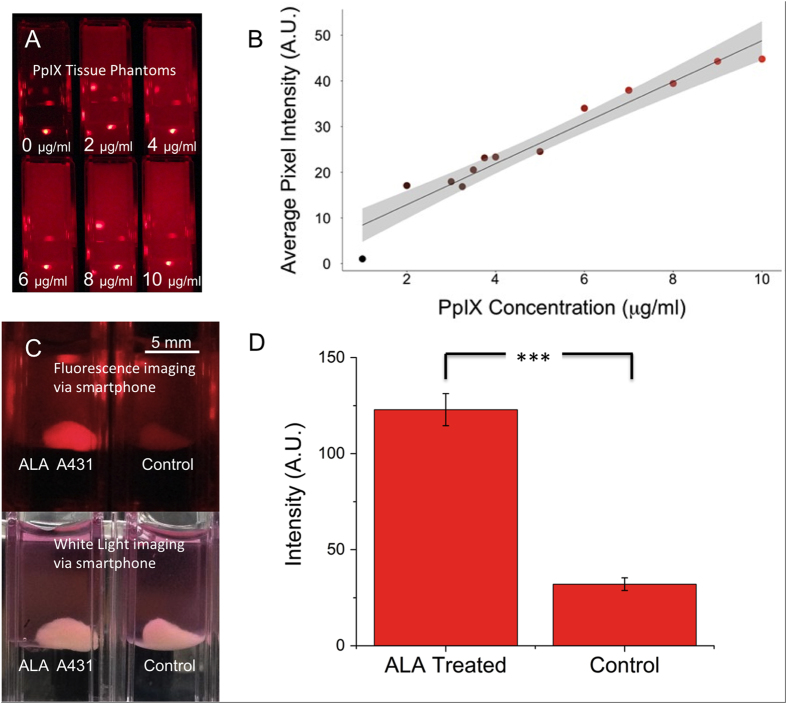
Smartphone-based quantitative PpIX fluorescence imaging in phantoms and A431 cell aggregates. Fluorescence images (**A**) of physiologically relevant concentrations of PpIX in phantoms was imaged using the smartphone setup shown in Fig. 5. Analysis using a custom routine developed via MATLAB image processing toolbox used to calculate concentration-dependent fluorescent intensity (**B**) In (**B**) the linear dependence of fluorescence signal over is shown (R^2^ = 0.9391) and 95% confidence interval (grey shading) are shown in (**C**) ALA treated (left) and control (right) A431 aggregate samples, imaged with smart phone imaging setup (top) and with normal lab lighting (white light, bottom). The visual contrast between ALA-treated and untreated tissue becomes clear when imaged via fluorescence. (**D**) Differential PpIX fluorescence emission of control and ALA treated centimetre-sized A431 aggregates, obtained using the smartphone-imaging setup is highly significant (*p < 10*^*–7*^).

We first evaluated the ability of the smartphone to detect PpIX fluorescence emission excited using the phone’s own built in LED flash with a blue-violet excitation filter. The output power in the appropriate spectral window however is not sufficient to excite PpIX emission to detectable levels, which is consistent with documentation of typical camera flash sources. Therefore we adapted a low-cost 405 nm LED array that could be mounted on the phone to provide an excitation source suitable for PpIX imaging, and fit a 660 nm band-pass filter (FWHM = 50 nm) into the central area of the device to serve as a PpIX emission filter (Fig. 5). Using this handheld imaging device, intensity detected on the phone camera chip was measured for tissue phantoms with tissue phantoms containing relevant concentrations of PpIX^35^,^36^, as shown in Fig. 6. Bright spots seen in Fig. 6A are specular reflections off of the cuvette surface and were not included in our analyses. Quantification of the measured fluorescent signal intensity increases linearly with increasing PpIX concentration roughly within a 95% confidence interval (grey shading on the plot). In Fig. 6C,D we further characterize imaging performance using cultured A431 tissues, in which cells were pre-treated with ALA prior to forming a pellet modelling a roughly centimetre sized lesion. The simple device used here is clearly able to differentiate the large contrast in fluorescence signal from non-ALA treated controls (p < 10^–7^), though the precise limits of sensitivity remain to be more extensively characterized with finer concentration gradients.

## Conclusion

In this study, we evaluate the capabilities of battery powered portable LED-based light sources for ALA-based PDT and tumour imaging. For treatment we show that a source powered by disposable alkaline batteries has sufficient stability for accurate dosimetry and achieves nearly complete cytotoxic response in 3D cultures of squamous carcinoma cells. The treatment device tested here was built of low-cost components with a total cost of less than $40 (the single most expensive part was the high-output LED itself, at $25). While performing PDT at realistic dose ranges under battery power does indeed present challenges, the outcomes of this study indicate that the rate of power output deterioration, with modest efforts at voltage regulation, is small relative to other well established sources of error in PDT dosimetry. In combination with smartphone based imaging for treatment guidance and monitoring, this indicates the feasibility of PDT as a viable cancer technology that can be deployed at remote and/or resource limited sites without electricity, a requirement that is very real in global health settings.

Integrating the results of the treatment and imaging components of this study, it is envisioned that a field healthcare worker, even in a remote setting, could administer ALA (in an oral suspension) and use the modified smartphone imaging device to provide tumour contrast for guiding subsequent oral light delivery using the higher intensity 635 nm source. The use of a smartphone-based imaging device further offers the potential for telemedicine capability, in which the PpIX tumour image at initial diagnosis and follow-up monitoring could be uploaded to a consulting physician at a remote site. Going forward, the established photosensitization strategy employed here, of PpIX accumulation induced by delivery of exogenous ALA (in a topical cream or oral ingestion in soda or juice), combined with the simplicity of the battery-powered devices, minimizes demand for highly skilled healthcare providers to administer the treatment. Simplicity of operation could be further enhanced by development of a custom smartphone app that would perform simple tumour size and dosimetry calculations from the image data.

While the simple battery powered device characterized here is suitable for in vitro PDT, and possibly treatment of superficial lesions, we envision that appropriate optics for light delivery would be incorporated for specific clinical PDT applications. For example, for oral cancer the LED source would likely need to be coupled to a multimode fibre with a light diffusing medium at the distal end to be placed in contact with the lesion in the oral cavity during irradiation. Given the power densities achieved here, the beam could be expanded and homogenized with small losses to accommodate larger treatment surfaces. There is also considerable room for improvement and optimization of the device packaging. The device used in this study was in an oversized housing for streamlined prototyping; however, all internal electronic components could fit in a 60 cubic inch box. Therapeutic performance could also be improved by the extensively documented observation that PDT efficacy can be enhanced by fractionated light delivery^39^,^40^. As a subsequent avenue for investigation, we hypothesize that in the battery-powered scenario, an appropriate fractionation schedule could be established for enhanced device performance due to the LED power output recovery effect described here, as well as the established photochemistry arguments for this approach.

In evaluating the capabilities of a consumer smartphone for imaging of PpIX fluorescence, the variation in intensity of PpIX of phantoms and cultured tissues measured here indicates that differential PpIX concentrations in tumour and normal tissue should be well within the imaging sensitivity of this approach. A logical extension of this finding is to conduct smart phone in vivo tumour imaging experiments and use additional optics (also available at low cost) to improve numerical aperture of the built in smartphone lens. The capability to measure photosensitizer fluorescence in this manner also lays the groundwork for a more comprehensive PDT platform, where LED device performance can be enhanced via smartphone-enabled online monitoring of PpIX uptake, localization and photobleaching, building on established methods^41^.

## Author Contributions

J.H., D.P.J., I.R., T.H. and J.P.C. designed research; J.H., D.P.J., G.M.C. and A.Z. conducted experiments; J.H., J.P.C., and S.A. designed/configured hardware; J.H., D.P.J. and J.P.C. wrote the main manuscript text and prepared figures; all authors reviewed and revised the manuscript.

## Additional Information

**How to cite this article**: Hempstead, J. *et al*. Low-cost photodynamic therapy devices for global health settings: Characterization of battery-powered LED performance and smartphone imaging in 3D tumor models *Sci. Rep.* **5**, 10093; doi: 10.1038/srep10093 (2015).

## Supplementary Material

Supplementary Information

## Figures and Tables

**Table 1:** Summary of performance power output and voltage stability performance during consecutive 15 minute runs on 3 AA batteries.

**Run #**	**Time (Minutes)**	**Voltage (V)**	**Power (mW)**	**% drop**	**% rebound**	***P***_**0**_**Δ*****t*** **(J)**	***1/2***(***P***_**0**_**+*****P***_***f***_**)Δ*****t*** **(J)**	**% error**
1	0	4.40	16.4		N/A	14.76	13.545	8.97
	15	4.17	13.7	16.46				
2	20	4.23	14.0		2.190	12.6	12.105	4.09
	35	4.14	12.9	7.86				
3	40	4.18	13.2		2.326	11.88	11.43	3.94
	55	4.10	12.2	7.58				
4	60	4.14	12.5		2.459	11.25	10.89	3.31
	75	4.07	11.7	6.40				
5	80	4.11	12.0		2.564	10.8	10.44	3.45
	95	4.03	11.2	6.67				
6	100	4.07	11.5		2.679	10.35	10.08	2.68
	115	4.01	10.9	5.22				
