# Supplementary material for: Low-cost photodynamic therapy devices for global health settings: Characterization of battery-powered LED performance and smartphone imaging in 3D tumor models
Source: Sci Rep. 2015 May 12;5:10093. doi: 10.1038/srep10093 (PMC4428052; doi:10.1038/srep10093)
Supplement: Supplementary Information [file srep10093-s1.pdf]

Supplemental Material

Low-cost photodynamic therapy devices for global health settings: Characterization of battery-powered LED performance and smartphone imaging in 3D tumor models

Joshua Hempstead,[a*] Dustin P. Jones,[a*] Abdelali Ziouche,[b] Gwendolyn M. Cramer,[a] Imran Rizvi,[c] Stephen Arnason,[a] Tayyaba Hasan,[c] Jonathan P. Celli[a]

[a]Department of Physics, University of Massachusetts, Boston, MA 02125, USA

[b] Laboratoire de Physique des Lasers, Université Paris 13, 93430 Villetaneuse, France

[c]Wellman Center for Photomedicine, Massachusetts General Hospital, Boston, MA 02114, USA

**Equal contribution*

Address correspondence to: Jonathan Celli (jonathan.celli@umb.edu)

Supplemental Figure 1

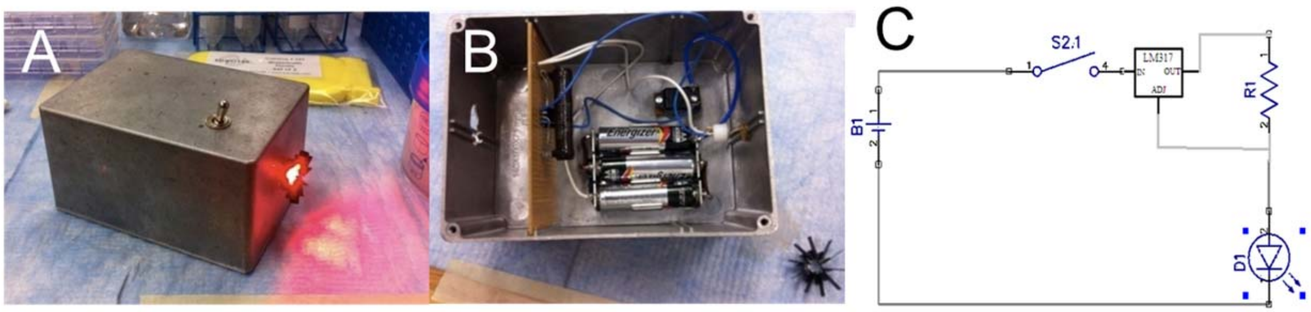

Supplemental Figure 1: LED irradiation device photographs and schematic. Photographs of the exterior **(A)** and interior **(B)** of the device used. The battery-powered circuit showing the battery source (B1), LED (D1), power resistor (R1), voltage regulator (LM317) and switch (S2.1) is depicted in **(C)**.

Supplemental Figure 2

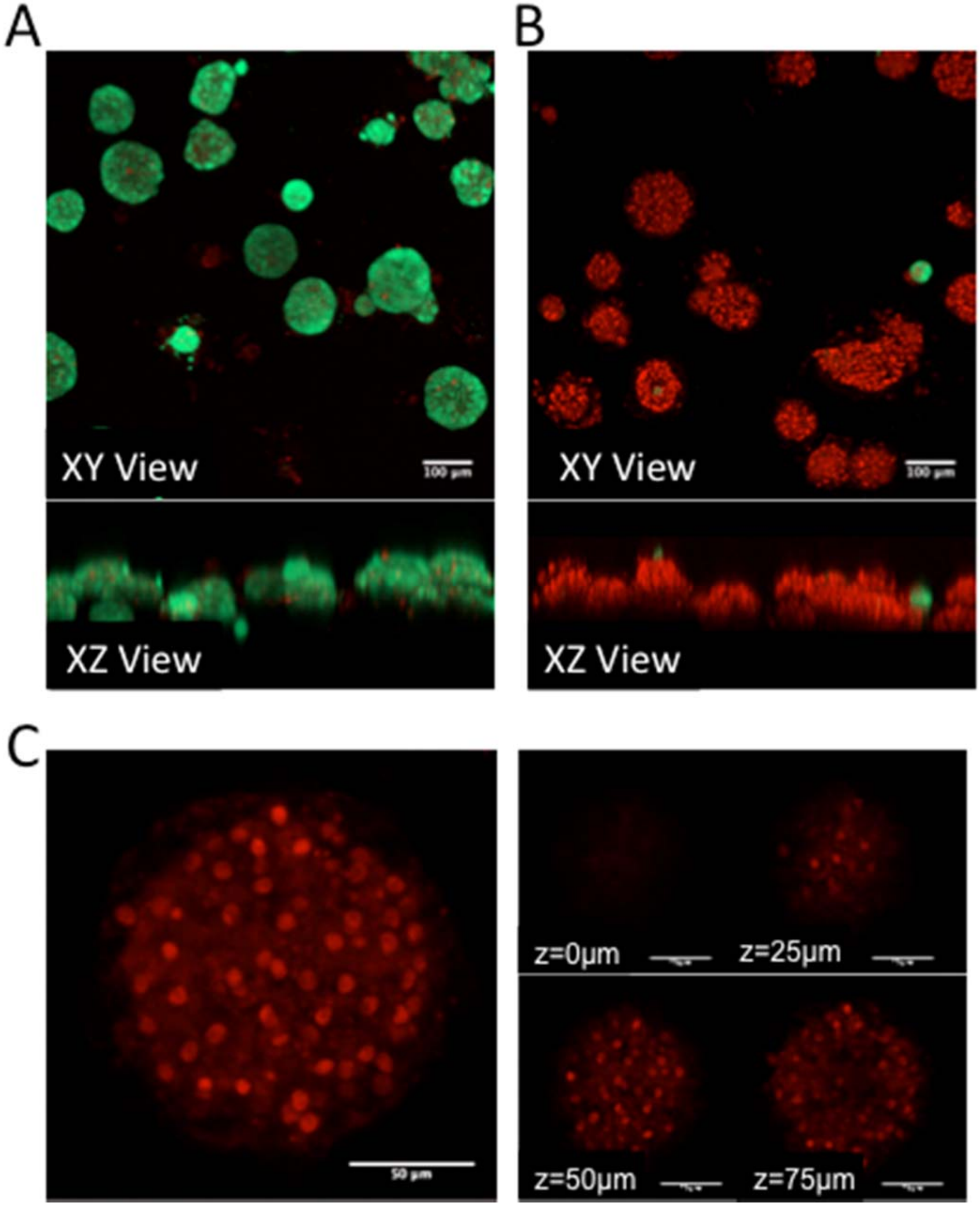

Supplemental Figure 2: Depth resolved cytotoxic response in 3D tumour models stained with calcein AM (green) and ethidium bromide (red) imaged via confocal microscopy. Large spatial fields showing XY (max intensity Z projection) and XZ views

of untreated cultures (A) and cultures treated with 80J/cm^2 ALA-PDT with light delivered via battery-powered device (B). 3D nodules are approximately spherical as previously characterized in other cell lines and have mean diameter (and equivalent depth) of 101 +/- 27 um with nearly complete cytotoxic destruction throughout. A zoomed in view of a representative 3D nodule in (C) shows depth resolved cytotoxic response in each optical section.
